# Open-chest versus closed-chest cardiopulmonary resuscitation in trauma patients: effect size is probably higher for penetrating injury

**DOI:** 10.1186/s13054-020-03372-w

**Published:** 2020-11-23

**Authors:** Romain Jouffroy, Benoît Vivien

**Affiliations:** 1 Intensive Care Unit, Ambroise Paré Hospital, Assistance Publique - Hôpitaux de Paris, Boulogne-Billancourt, France; 2 SAMU de Paris, Service d’Anesthésie-Réanimation, Hôpital Universitaire Necker - Enfants Malades, APHP, Centre, Assistance Publique - Hôpitaux de Paris, and Université de Paris, Paris, France

To the Editor,

Endo et al. [1] recently reported that compared to closed-chest cardiopulmonary resuscitation (CCCPR), open-chest cardiopulmonary resuscitation (OCCPR) was associated with significantly higher survival at hospital discharge in severe trauma patients with signs of life upon emergency department arrival.

The authors should be congratulated for their very interesting study in the utmost important field of traumatic cardiac arrest. Nevertheless, we believe that some points of their study should be pointed out.

Endo et al. used a propensity score matching analysis to mimic a randomized control trial, and the readers should be aware that this methodology reduces the differences according to the type of injury. Indeed, international guidelines for cardiopulmonary resuscitation (CPR) recommend, beyond symptomatic CPR, the etiological treatment of reversible causes of cardiac arrest, which are summarized by the 4H and 4T mnemonic tool (i.e., hypovolemia, hypoxemia, hypo/hyperkalemia, hypothermia, toxic, tamponade, pneumothorax, and pulmonary/coronary thrombosis) [2, 3]. However, propensity score matching reduces the differences between blunt and penetrating injury groups, for whom cardiac arrest etiological treatment present major differences. First, penetrating injuries result in a higher cardiac arrest proportion requiring an open-chest intervention that may fully explain the relative weight of the variable, “number of trauma surgeons in a hospital,” on outcomes observed in Endo et al. study [1]. Second, blunt trauma patients generally present multiple injuries, many of which include brain trauma, which could dramatically impair prognosis whenever any injury occurs that is accessible to treatment by OCCPR. Finally, the propensity score matching does not include two major cardiac arrest outcome determinants: the no-flow and the low-flow durations [4, 5].

Beyond these considerations, we agree with Endo et al. [1], that the use of OCCPR should be considered more frequently for trauma patients presenting with cardiac arrest upon hospital arrival, especially those with penetrating injury.

## Data Availability

Not applicable.
